# Quality control and external quality assessment for the independent clinic-based evaluation of point-of-care testing to detect *Chlamydia trachomatis*, *Neisseria gonorrhoeae* and *Trichomonas vaginalis* in eight countries

**DOI:** 10.1186/s12879-024-09057-x

**Published:** 2024-02-29

**Authors:** Mark Shephard, Susan Matthews, Kelly Andrewartha, Wayne Dimech, Liza Cabuang, Christopher Barbara, Xiang-Sheng Chen, Maddalena Cordioli, Amina Hançali, Ting-Ting Jiang, Ranmini Kularatne, Stephanie Meli, Etienne Muller, Hicham Oumzil, Valeska Padovese, Angela Sandri, Silver Vargas, Graziella Zahra, Magnus Unemo, Karel Blondeel, Igor Toskin

**Affiliations:** 1 International Centre for Point-of-Care Testing, Flinders University, Bedford Park, South Australia Australia; 2 National Serology Reference Laboratory, Australia, Fitzroy, Victoria Australia; 3 Pathology Department, Mater Dei Hospital, Msida, Malta; 4 Chinese Academy of Medical Sciences and Peking Union Medical College Institute of Dermatology, National Center for STD Control, Nanjing, China; 5 Infectious Diseases Section, Department of Diagnostics and Public Health, University of Verona, Verona, Italy; 6 STIs Laboratory, Department of Bacteriology, Institut National d’Hygiene, Ministry of Health- Morocco, Rabat, Morocco; 7 Centre for HIV & STI, National Institute for Communicable Diseases, Sandringham, South Africa; 8 Point-of-Care Testing Committee, Department of Pathology, Mater Dei Hospital, Msida, Malta; 9 Pedagogy and Research Unit of Microbiology, Faculty of Medicine and Pharmacy, Mohammed V University –Rabat, Virology Department, Institut National d’Hygiène MoH, Rabat, Morocco; 10 Genito-Urinary Clinic, Department of Dermatology and Venereology, Mater Dei Hospital, Msida, Malta; 11 Department of Diagnosis and Public Health, Section of Microbiology, University of Verona, Verona, Italy; 12 Centro de Investigación Interdisciplinaria en Sexualidad SIDA y Sociedad, Universidad Peruana Cayetano Heredia, Lima, Peru; 13 Molecular Diagnostics Infectious Diseases, Department of Pathology, Mater Dei Hospital, Msida, Malta; 14 WHO Collaborating Centre for Gonorrhoea and Other STIs, Department of Laboratory Medicine, Faculty of Medicine and Health, Örebro University, Örebro, Sweden; 15 Department of Sexual and Reproductive Health and Research, World Health Organization, Geneva, Switzerland; 16 Faculty of Medicine and Health Sciences, Ghent University, Ghent, Belgium

**Keywords:** Point-of-care testing, Sexually transmitted infections, GeneXpert, Training, Quality, Concordance, Test errors

## Abstract

**Background:**

Sexually transmitted infections caused by *Chlamydia trachomatis* (CT), *Neisseria gonorrhoeae* (NG) and *Trichomonas vaginalis* (TV) remain significant global health problems. The World Health Organization (WHO) has recently conducted a multi-faceted, multi-country validation study (ProSPeRo), which included an evaluation of the Xpert CT/NG and Xpert TV assays on the GeneXpert system (Cepheid, Sunnyvale, Ca., USA) in clinic-based settings across eight countries. To support the study, a training and quality management system was implemented and evaluated.

**Methods:**

A comprehensive training program for the study was developed. Quality control (QC) and external quality assessment (EQA) samples were provided by an accredited quality assurance provider. QC testing was conducted at 14 point-of-care testing (POCT) clinics, while EQA samples were tested by the POCT sites and a reference laboratory supporting each clinic.

**Results:**

For QC testing, concordance with the expected results for CT and NG was > 99% and rates of unsuccessful tests were < 4%. For TV testing, concordance was similar (97%), but rates of unsuccessful tests were high (18%), particularly in the ‘TV negative’ sample. For EQA testing initially conducted in 2018, concordance was 100% for CT and NG, and 90% for TV for the reference laboratory group (which used non-GeneXpert systems). Concordance for the POCT group was also high (> 94%) for all analytes, but this cohort (which used GeneXpert systems) exhibited a high rate of unsuccessful TV tests. All but one of these unsuccessful tests was subcategorised as ‘invalid’.

**Conclusions:**

The high level of concordance for QC and EQA testing confirm that the trained operators at the POC clinical sites were competent to conduct POC testing and that the training and quality systems implemented for the ProSPeRo study were effective. The quality materials used were satisfactory for CT and NG but exhibited poor performance for TV testing on the GeneXpert system. The WHO should continue to work with industry and EQA providers to provide improved materials that are reliable, stable and cost effective for quality management, as it seeks to rollout molecular-based STI POCT in non-laboratory-based settings.

**Trial registration:**

Ethics approval to conduct the ProSPeRo study was granted by the WHO Ethics Review Committee.

## Background

In 2021, the World Health Organization (WHO) estimated that over 367 million people aged 15 to 49 globally were infected each year with one of three curable sexually transmitted infections (STIs): *Chlamydia trachomatis* (CT), 128 million; *Neisseria gonorrhoeae* (NG), 82 million; and *Trichomonas vaginalis* (TV) 156 million [1]. These concerning figures were similar to those reported by the WHO in 2016, indicating that these STIs remain a significant contemporary health problem [2].

All three STIs are treatable but, if left inappropriately treated or undetected, they can result in long-term complications such as pelvic inflammatory disease, tubal infertility and ectopic pregnancy in women [3]. Furthermore, antimicrobial resistance in NG has increased worldwide and concerns that gonorrhoea may become untreatable in certain circumstances have been raised [4].

Early detection of these STIs with resultant rapid treatment is crucial, particularly where the prevalence of disease is greatest, such as in low-and-middle income countries (LMIC) and in young people, sex workers, men who have sex with men, First Nations communities and people living in rural and remote settings *inter alia* [5].

The use of point-of-care testing (POCT) for STIs, where testing is conducted on-site during the patient consultation, has the potential to deliver rapid test results followed by timely initiation of appropriate treatment, thereby enhancing the opportunity to interrupt the cycle of transmission in a community setting. The advent of molecular-based POCT platforms with laboratory-equivalent analytical performance is revolutionising the field of STI diagnosis, particularly in primary care settings [5].

The WHO has recently updated and published target product profiles, which highlight the attributes and desirable operational and analytical performance characteristics of the ‘ideal’ POCT assay for selected STIs [6].

From 2013 to 2015, a world-first randomised controlled trial of the GeneXpert system (known as Test Treat ANd GO [TTANGO]) was conducted in a primary care setting in Australia (among remote First Nations communities); this landmark study demonstrated that the molecular-based technology, in this setting, was accurate when compared to parallel laboratory testing, reduced time to treatment, increased treatment uptake and was well accepted by health professionals (mainly remote area nurses) conducting the test [7–10].

Since 2017, the WHO has undertaken a multi-country validation study known as ProSPeRo (the Project on Sexually Transmitted Infection Point-of-Care Testing established by the Sexual and Reproductive Health and Research Department of WHO). The study involved several discrete arms (reported elsewhere in this Supplement), including the present study which evaluated the Xpert CT/NG and Xpert TV assays on GeneXpert systems (Cepheid, Sunnydale, California, USA) in three different patient populations at clinic-based POCT sites across eight countries – Australia, China, Italy, Guatemala, Malta, Morocco, Peru and South Africa. The study had two major aims: (i) to determine the performance characteristics of the Xpert CT, NG and TV POCT on the GeneXpert systems compared to that of the best available nucleic acid amplification tests (NAAT) conducted at an in-country reference laboratory or, if not available in-country, at an international reference laboratory and (ii) to assess the minimal operational characteristics and acceptability of the GeneXpert test system for Xpert CT, NG and TV POCT to health users and health professionals.

To support the CT, NG and TV arms of the ProSPeRo study, a training program and a quality management system were developed and integrated into the broader study framework [11, 12]. For the quality testing component, samples for quality control (QC) and external quality assessment (EQA) were provided to each clinic-based POCT site. EQA samples were also provided to the reference laboratories supporting the clinics in the study. This article describes the establishment of the training and quality systems used in this arm of the study and provides commentary on their implementation and lessons learned for future integration into the primary care sector.

## Methods

### Ethics approval and consent to participate

Ethics approval for the core protocols of the CT, NG and TV arms of the ProSPeRo study was obtained by the WHO Ethics Review Committee (ERC). Locally adapted site-specific protocols were approved by the respective local ethics committees and by ERC.

### Countries and sites involved

Eight countries participated in the study: Australia, China, Guatemala, Italy, Malta, Morocco, Peru and South Africa (randomly reported hereafter as countries A-H). The STI tests performed in the study were at the discretion of each participating country and were dependent on the populations tested. In Australia, Morocco and South Africa, STI testing was conducted on vaginal swab specimens from asymptomatic women ‘at risk’ of contracting STIs; in China and Guatemala, on swabs from women with vaginal discharge; and, in Italy, Malta and Peru, on urine and extragenital samples (anorectal and pharyngeal swabs) from men who have sex with men (MSM). Four countries tested for all three analytes, three countries performed CT and NG but not TV, and one country performed TV only. The number of participating POCT clinics in each country is also shown in Table 1. In Australia, three remote POCT clinics were initially engaged, but two withdrew early due to significant unplanned staffing issues. One remote clinic from Northern Australia completed the study.

**Table 1:** Sexually transmitted infection tests performed by reference laboratories and POCT clinics in participating countries

Country	Tests performed by Reference Laboratory		System used by Reference Laboratory	Tests performed by POCT clinics		System used by POCT clinics	Number of participating POCT clinics
	CT/NG	TV		CT/NG	TV		
Australia	NA	√	Hologic Panther	NA	√	GeneXpert	3
China	√	√	Roche Cobas 4800 (CT/NG); Local test (TV)	√	√	GeneXpert	3
Italy	√	NA	Roche Cobas 4800 (CT/NG)	√	NA	GeneXpert	1
Guatemala	√	√	Hologic Panther	√	√	GeneXpert	2
Malta	√	NA	Hologic Panther	√	NA	GeneXpert	1
Morocco	√	√	Hologic Panther	√	√	GeneXpert	1
Peru	√	NA	Hologic DTS 400	√	NA	GeneXpert	2
South Africa	√	√	Hologic Panther	√	√	GeneXpert	1

*NA not applicable, test not performed by that country, *POCT* point-of-care testing, *CT *Chlamydia trachomatis, *NG *Neisseria gonorrhoeae, *TV *Trichomonas vaginalis

### Molecular-based STI diagnostic systems used in ProSPeRo

The POCT clinics performed STI testing for CT, NG and/or TV on the GeneXpert system using a dual test cartridge for simultaneous qualitative detection of CT/NG and a single test cartridge for the qualitative detection of TV. The reference laboratories used either the Aptima Combo 2 assay (for CT and NG) and Aptima TV assay on the Panther system or Tigris DTS 400 system (Hologic, Massachusetts, USA) or the Cobas assay on the Cobas 4800 system for CT/NG and Cobas 6800 system for TV (Roche, Switzerland) (Table 1) [13]. The reference laboratory in Australia changed its test system for TV during the study from the Panther to the Cobas. For Guatemala, Malta and Morocco, samples were tested by an international STI reference laboratory in Sweden (the WHO Collaborating Centre for Gonorrhoea and other STIs) using the Panther system.

### Training system

A comprehensive training program for the study, consistent with the most recent National Pathology Accreditation Advisory Council (NPAAC) Requirements for Point-of-Care Testing in Australia 2021 [14] was developed by the Flinders University International Centre for Point-of-Care Testing (ICPOCT).

**Fig. 1:**
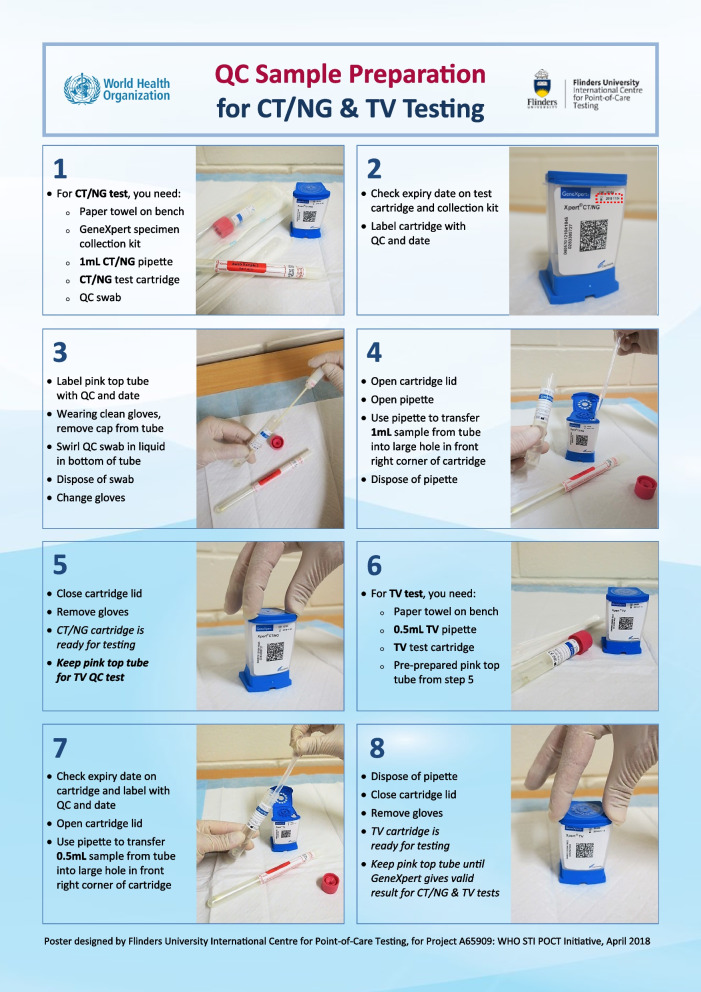
Poster on testing quality control samples provided to participants in this study (Used with permission. Copyright© Flinders University International Centre for Point-of-Care Testing, 2018)

The training package included information on the GeneXpert system, such as basic operation, method principle, the testing cartridge, how to conduct a patient, QC and EQA test, and interpretation of test results. A series of step-by-step posters and short YouTube videos visually summarised the performance and reporting of patient, QC and EQA tests (Fig. 1). (https://www.youtube.com/watch?v=V4wBkS80peY and https://www.youtube.com/watch?v=1qKvDlY_GMk).

Operator training was delivered through face-to-face sessions conducted on-site in participating countries. In Australia, training was presented by an ICPOCT scientist, and in other countries by a Cepheid representative (general operation of the GeneXpert system) and by WHO study team members (QC and EQA components). In Guatemala and China, the YouTube videos prepared by ICPOCT were translated live during the delivery of training. Local research teams also organised refresher and practical QC and/or EQA testing at initiation of the studies. In Australia, operators completed a written competency assessment and practical assessment using QC and/or EQA materials.

### Quality management system

The National Serology Reference Laboratory, Australia [NRL], was contracted by the WHO to provide QC and EQA samples for the ProSPeRo study. NRL is accredited to ISO 17043 as a proficiency testing scheme provider that produces quality assurance programs to support the quality of infectious disease testing both in Australia and internationally. The NRL had previously provided EQA materials for the CT/NG component of the TTANGO program [8].

### Production of QC and EQA samples by NRL

NRL produced positive and negative QC and EQA materials for the ProSPeRo study, using procedures previously validated for CT and NG [15].

The positive quality samples for CT, NG or TV were prepared by initially suspending known positive materials (at defined concentrations or reactivity) into molecular-grade phosphate buffered saline (PBS). Individual swab samples were then inoculated with 50 μL of selected material (see Table 2) and left to dry for 4 hours in a Class 2 biosafety cabinet. All swabs contained baseline human cells (Huh-7) at a concentration of 2 × 10^5^ cells/mL. The negative quality samples contained PBS only. All swabs were transported to all participating sites at ambient temperature (25-35 °C) and the materials stored between 2 and 8 °C prior to testing.

**Table 2:** Composition of the swabs prepared by NRL for quality control (QC) and external quality assessment (EQA) testing^b^

Composition	CT only	NG only	CT/NG Duo	TV only	Negative
Concentration	4 × 10^4^ DNA copies/mL	4 × 10^4^ DNA copies/mL	4 × 10^5^ DNA copies/mL of both CT and NG	1/500 dilution of liquid culture^a^	PBS only, no organisms
Internal control (baseline)	Uninfected Huh-7 cells, 2 × 10^5^ cells/mL				

The QC samples consisted of the CT/NG Duo and the TV only swab samples. The EQA samples consisted of any combination of the five manufactured samples

*CT *Chlamydia trachomatis, *NG *Neisseria gonorrhoeae, *TV *Trichomonas vaginalis

^a^ designed to provide a target cycle threshold of between 30 and 35

^b^ Previous studies showed that (i) the concentrations provided in these samples showed an insignificant variation in Ct value when stored at -20 °C or 23-25 °C and (ii) the material was stable for at least 2 years when stored at 2-8 °C [15, 16]. According to the manufacturer, the TV material provided for this study was produced following the same process as described for CT/NG, however no validation data was provided

### Testing of QC samples

For QC testing, participating POCT clinics were each provided with a set of 15 swabs that were ‘CT positive, NG positive and TV negative’ (QC 1) and 15 swabs that were ‘CT negative, NG negative and TV positive’ (QC 2). The testing of a negative QC using molecular-based methods is important in instances where environmental or amplicon contamination may be an issue.

QC swabs were prepared for testing by inoculation into a GeneXpert swab transport reagent tube. Using a transfer pipette, an aliquot of this medium (1 mL for CT/NG and 0.5 ml for TV) was loaded into a GeneXpert test cartridge, as per manufacturer’s instructions. QC swabs were tested during operator training (positive and negative) and thereafter once monthly (positive and negative) for the duration of patient testing. This frequency of testing aligned with the minimum requirements for QC testing promulgated in national recommendations for POC testing in Australia and used in the TTANGO POC testing network [14, 16].

### Reporting of QC results and feedback provided

Participant operators recorded the site and date of testing and the GeneXpert result for each QC sample on the QC form provided. Four options were provided for result entry – ‘not detected’, ‘detected’, ‘unsuccessful’ or ‘not applicable’. If an ‘unsuccessful’ result was reported, operators were encouraged to repeat the test and complete a ‘comments’ box documenting additional information on error code. The GeneXpert does allow for further categorisation of ‘unsuccessful’ tests as ‘invalid’, ‘error’ or ‘no result’ (see next section). The ‘not applicable’ box was ticked if the site did not perform either the CT/NG or TV test (according to Table 1).

Completed QC result sheets were emailed to ICPOCT and/or the ProSPeRo study organisers. A QC action flowchart was provided as an interpretive aid for operators. A QC feedback report also summarised the results submitted by each individual site for the past 3 months and highlighted whether returned results were ‘consistent’ (concordant) or ‘inconsistent’ (discordant) with the expected results. The results of individual sites were also peer-reviewed against other participating sites.

### Testing of EQA samples

For EQA testing, each participating clinic and reference laboratory were provided with two sets of five swab samples at the start of the study (2018). Set A samples were tested at the commencement of the study and Set B samples were tested at least 6 months post the start of the study. The two EQA sets contained random within and between combinations of both positive and negative swab samples for CT, NG and TV.

EQA swabs were prepared and tested using the same process as described for the QC samples. The expected results for the EQA swabs were unknown to the operator at the time of testing.

### Reporting of EQA results and feedback provided

Each site was provided with an EQA result sheet for Set A and B. Again, four options for result entry were provided – ‘not detected’, ‘detected’, ‘unsuccessful’ or ‘not applicable’ – with a comments box for further information on error code. An EQA feedback report also summarised the results submitted for the five samples tested in each set, noted whether they were ‘consistent’ or ‘not consistent’ with expected results, and provided peer review feedback on the site’s performance compared to other participants.

Due to a manufacturing issue with the TV component of the EQA material (see Results section), a second set of EQA materials (with different combinations of positive and negative samples) were made by NRL in 2019 and distributed to all countries (except China).

### In-built quality checks on the GeneXpert system

In addition to QC and EQA testing, the GeneXpert system has in-built quality checks. These internal quality checks include a) a sample processing control (SPC) to verify adequate processing of the target bacteria (for CT and NG) or protozoan parasite (TV), b) a sample adequacy control (SAC) to confirm whether the sample contains human DNA and c) a probe check control (PCC) to verify reagent rehydration, PCR tube filling, probe integrity and dye stability. An ‘invalid’ result indicates that the SPC and/or the SAC failed. An ‘error’ result indicates that the PCC failed, and the assay was aborted. A ‘no result’ flag indicates that insufficient data were collected, most likely due to the operator stopping a test that was in progress or a power failure occurring.

## Results

### Staggered implementation of the ProSPeRo study

The patient testing phase for different countries was scheduled to be 12-months’ duration but, owing to the global COVID-19 pandemic, the program implementation was staggered across an almost three-year time frame from mid-2018 to 2021.

### Training

Across the participating countries, 15 operators from the POCT clinics were trained as primary GeneXpert operators, while 10 operators were trained from associated reference laboratories (range one to five operators per country). 

### QC results

A summary of the valid QC tests and results performed on the GeneXpert at the 14 POCT sites across the participating countries is provided in Table 3.

**Table 3:** Number of valid QC tests performed for CT, NG and TV by POC testing sites

Country	Site	QC 1: Expected result			QC 2: Expected result		
		CT Pos	NG Pos	TV Neg	CT Neg	NG Neg	TV Pos
A	1	5	5	–	5	5	–
B	2	–	–	1	–	–	1
B	3			1			1
B	4	–	–	–	–	–	–
C	5	7	7	–	3	3	–
C	6	8	8	–	8	8	–
D	7	12	12	–	12	12	–
E	8	7	7	4	6	6	6
F	9	1	1	–	–	–	1
F	10	1	1	–	–	–	1
G	11	5	5	4	4	4	5
H	12	8	8	7	7	7	8
H	13	9	9	4	7	7	9
H	14	5	5	4	4	4	5
Total results		68	68	25	56	56	37
Number (%) discordant		0	1 (1.5%)	0	0	0	2 (5.5%)

‘-‘means country /POC site did not test for this analyte

*QC* quality control, *CT *Chlamydia trachomatis, *NG *Neisseria gonorrhoeae, *TV *Trichomonas vaginalis, *POC* point of care, ‘*Pos*’ expected result: detected, ‘*Neg*’ expected result: not detected

Combining the data for CT and NG testing on the two QC samples provided, overall concordance with the expected results was 100% for CT and 99% (135/136) for NG, with just 3.7% (5/136) of tests performed by the POCT sites reported as ‘unsuccessful’. For TV, overall concordance with the expected result was 96.8% (60/62). However, 18% of TV tests (13/72) attempted for QC 1 and QC 2 were initially ‘unsuccessful’, the majority of which (10/13) were recorded for QC 1 (TV negative). Nine of the 13 ‘unsuccessful’ tests were categorised as ‘invalid’.

### EQA results

EQA samples from Set A (2018) were tested by reference laboratories from four countries (A, C, D and E). POCT sites from seven countries (A, B, C, D, E, F and G) tested the EQA samples for CT and NG, but only four countries (B, E, G and F) tested these for TV. Country H used an in-house EQA material rather than that provided by NRL (and their results were not included in the data analysis). The international STI reference laboratory in Sweden did not participate in the NRL EQA testing program.

The combinations of expected test results for Set A (2018) samples are shown in Table 4, together with the number of valid EQA results reported for each sample.

**Table 4:** Summary of number of valid results from EQA testing for Set A, 2018

Sample	Expected Result	Reference Laboratories	POC Testing Clinics
Sample 1	CT Neg	4	7
	NG Pos	4	7
	TV Pos	2	3
Sample 2	CT Pos	4	7
	NG Pos	4	7
	TV Neg	2	4
Sample 3	CT Neg	4	7
	NG Neg	4	7
	TV Neg	2	2
Sample 4	CT Pos	4	8
	NG Neg	4	8
	TV Neg	2	4
Sample 5	CT Neg	4	8
	NG Neg	4	8
	TV Pos	2	4
Total		50	91

*EQA* External Quality Assessment, *CT *Chlamydia trachomatis, *NG *Neisseria gonorrhoeae, *TV *Trichomonas vaginalis, *POC* Point of care, ‘*Pos*’ expected result: detected, ‘*Neg*’ expected result: not detected

A total of 50 valid EQA results for Set A (2018) were reported for the three analytes by the reference laboratory group. The laboratories achieved 100% concordance (40/40) for both CT and NG results and no ‘unsuccessful’ tests were reported for these analytes. For TV testing, concordance was 90% (9/10), with one ‘not detected’ EQA result being reported as TV ‘positive’ by country D’s reference laboratory. No ‘unsuccessful’ TV tests were reported by the reference laboratory group (all of which used non-GeneXpert systems).

A total of 91 valid EQA results were reported for the three analytes by the POCT clinics. The clinics achieved 97% (36/37) concordance for CT and 100% concordance (37/37) for NG results. Three ‘unsuccessful’ tests were reported for both CT and NG, but these were not repeated or categorised further. On initial testing of the TV EQA samples by the four participating countries, there were 10 valid and 20 ‘unsuccessful’ tests reported (66% [20/30] fail rate). Repeat analysis of seven of these 20 initial ‘unsuccessful’ tests produced a further seven valid test results. Concordance for the 17 valid TV EQA test results was 94% (16/17), with one site reporting a TV ‘not detected’ result on ‘positive’ sample 5. Twelve (60%) of the 20 ‘unsuccessful’ tests reported for set A were categorised as ‘invalid’, while no further information was provided for the remaining unsuccessful tests.

Due to participant concerns relating to the TV component of the initial EQA material from Set A (2018), only one POCT site (country A) and two reference laboratories (country A and C) submitted results for Set B (2018). All three sites achieved 100% concordance with the expected results for CT and NG, but none of these sites tested Set B samples for TV.

## Discussion

There is now an irrefutable body of evidence that POCT, with the support of governments and political will, can be a life changing tool in improving access to pathology testing and providing clinical, cultural, operational and economic benefits for patients with chronic, acute and infectious disease living in rural and remote communities globally [17–20]. The WHO has recognised the value of POCT and, in complementing the present multi-country validation study and the broader ProSPeRo study, the WHO has also made a ‘call to action’ for the use of (molecular-based) STI POCT to be integrated into health systems as a viable means of addressing the ongoing global STI epidemic [21].

Operator training and quality management are two core elements that underpin the success and sustainability of such effective POCT networks [11, 12]. Ongoing training provides a practical solution to reducing the impact of staff turnover in a POCT network, while the provision of bespoke options for training delivery and a flexible range of training resources for health professionals are essential components of contemporary POCT networks. In this study, adaptable delivery formats including face-to-face sessions involving participating scientists as well as an industry representative, and YouTube videos that were translated live during training were used effectively and led to GeneXpert operator competency, while training resources included hard copy and on-line materials, posters and interpretive aids.

A sound quality management system is needed to ensure the analytical quality of POCT can be continuously monitored and patient safety is not compromised. Without sound analytical quality, equivalent to that expected of a laboratory, a POCT network will not be sustainable. There are now many examples in the literature to show that POCT can meet current laboratory benchmarks/analytical goals for quality [22, 23]. A key component of quality surveillance is the provision of stable and reliable materials for QC and EQA testing.

In this study, a globally recognised and accredited quality assurance provider and WHO Collaborating Centre, (NRL), was commissioned to provide the materials for QC and EQA testing for molecular-based STI testing on GeneXpert systems. For the CT and NG component of QC testing, concordance with the expected results at the POCT clinical sites was very high (> 99%) and rates of ‘unsuccessful’ tests were very low (< 4%), indicating the QC material provided performed well for these analytes. For TV testing, concordance at these sites was high (> 96%), but rates of ‘unsuccessful’ tests in the GeneXpert were unacceptably high (18%), particularly in the ‘TV negative’ sample.

For EQA testing on Set A 2018 samples, concordance with expected results was 100% for CT, 100% for NG, and 90% for TV for the reference laboratory group (which used non-GeneXpert systems) and there were no ‘unsuccessful’ EQA tests reported. Concordance for the POC testing group was similarly high (> 94% for all analytes), but this cohort (which exclusively used GeneXpert systems) exhibited a high rate of ‘unsuccessful’ tests of 60% (20/30) for Set A (2018). Where these ‘unsuccessful’ TV tests were able to be subcategorised, all but one of these tests were ‘invalid’, indicating that the SPC and/or the SAC failed.

The high level of concordance observed for QC and EQA testing for both CT/NG and TV (all > 94%) confirm that the trained operators at the POCT clinical sites were competent to conduct POCT for these analytes. The high rates of unsuccessful tests observed for TV QC and EQA testing were specific to the GeneXpert test system used by the POCT clinical sites and were most likely due to a manufacturing issue.

While there are a range of quality testing products commercially available for CT and NG, at the commencement of this study there were no viable options available for TV QC and EQA. Therefore, a new source material was prepared by NRL for this study and used to manufacture both the QC and EQA components of this study. Unfortunately, the high rates of ‘unsuccessful’ TV tests on the GeneXpert system used at POCT clinical sites impacted the study negatively, as competent POCT operators at these sites lost trust in the material and in their ability to confidently perform quality testing.

There are several plausible reasons for the poor performance exhibited by the QC and EQA materials for TV, even though the swabs were manufactured in the same way as CT and NG, following NRL’s validated methods. The difference in organism type (i.e. CT and NG are both bacteria and TV is a protozoan) may have contributed to the increased rate of unsuccessful TV tests. Protozoal trichomonas has a more complex structure (such as presence of organelles) which may contain enzymes that can further degrade (any) nucleic acids. Other alternative explanations to account for the high rate of invalid TV results could potentially include (i) the concentration of human cells provided as baseline for all swabs was approaching the limit of detection for the SAC check when tested in the TV assay only (noting a SAC fail can only occur in a TV negative sample), or (ii) additional stabilisers needed to be added to the prepared material to prevent any degradation of human cells. Further investigations by NRL to resolve the rate of unsuccessful TV results in the GeneXpert system are on-going.

Consideration should be given to a training program for national and regional STI reference laboratories to enable them to produce QC/EQA materials at affordable prices for POCT sites in their proximity. Ideally, POCT sites particularly in LMICs, should not be dependent on EQA/QC materials produced and transported at high cost from HICs. The WHO needs to continue to work with industry and quality assurance providers to provide materials with such specifications, as it seeks to rollout molecular-based STI POCT to the developing world.

## Conclusion

The GeneXpert system is a sophisticated molecular-based testing platform for STI testing and has proven robust and accurate for CT and NG POCT in the hands of non-laboratory trained operators in the primary care setting in Australia [7]. However, the present study reinforces that, for a sustainable and scalable POCT network in a multi-country setting, it is crucial that reliable, stable and cost-effective materials for quality testing are available to support the widespread use of this test system.

## Data Availability

The data set analysed during the current study is available from the ProSPeRo study co-ordinators (KB and IT) upon reasonable request.
